# Brake response time before and after total knee arthroplasty: a prospective cohort study

**DOI:** 10.1186/1471-2474-11-267

**Published:** 2010-11-18

**Authors:** Michael C Liebensteiner, Michaela Kern, Christian Haid, Conrad Kobel, David Niederseer, Martin Krismer

**Affiliations:** 1 Department of Orthopaedic Surgery, Innsbruck Medical University, Innsbruck, Austria; 2 Department of Internal Medicine, Innsbruck Medical University, Innsbruck, Austria; 3 Department of Medical Statistics, Informatics and Health Economics, Innsbruck Medical University, Innsbruck, Austria; 4 Department of Sports Medicine, Prevention and Rehabilitation, Paracelsus Medical University Salzburg, Salzburg, Austria

## Abstract

**Background:**

Although the numbers of total knee arthroplasty (TKA) are increasing, there is only a small number of studies investigating driving safety after TKA. The parameter 'Brake Response Time (BRT)' is one of the most important criteria for driving safety and was therefore chosen for investigation.

The present study was conducted to test the hypotheses that patients with right- or left-sided TKA show a significant increase in BRT from pre-operative (pre-op, 1 day before surgery) to post-operative (post-op, 2 weeks post surgery), and a significant decrease in BRT from post-op to the follow-up investigation (FU, 8 weeks post surgery). Additionally, it was hypothesized that the BRT of patients after TKA is significantly higher than that of healthy controls.

**Methods:**

31 of 70 consecutive patients (mean age 65.7 +/- 10.2 years) receiving TKA were tested for their BRT pre-op, post-op and at FU. BRT was assessed using a custom-made driving simulator. We used normative BRT data from 31 healthy controls for comparison.

**Results:**

There were no significant increases between pre-op and post-op BRT values for patients who had undergone left- or right-sided TKA. Even the proportion of patients above a BRT threshold of 700 ms was not significantly increased postop. Controls had a BRT which was significantly better than the BRT of patients with right- or left-sided TKA at all three time points.

**Conclusion:**

The present study showed a small and insignificant postoperative increase in the BRT of patients who had undergone right- or left-sided TKA. Therefore, we believe it is not justified to impair the patient's quality of social and occupational life post-surgery by imposing restrictions on driving motor vehicles beyond an interval of two weeks after surgery.

## Background

Total knee arthroplasty (TKA) is a demonstrably successfully procedure being performed increasingly often [1-3] in patients with osteoarthritis of the knee [3-5]. Particularly the elderly are dependent on their driving abilities in order to execute activities of daily living [6]. Those scheduled for TKA frequently wish to know when they can resume driving after the operation. Brake response time (BRT) (also referred to as driving reaction time by some authors [7-9]) is one of the most important factors responsible for driving safety [9,10]. BRT has been investigated in the context of various orthopedic treatments [6,8,9,11-13].

In two studies concerning the effects of TKA on BRT [7,10] the following was reported: Spalding et al. [7] measured the BRT of patients receiving TKA and reported recovery of BRT after 8 weeks following TKA performed on the right leg. Pierson et al. [10] investigated 31 patients before and after TKA, found significantly reduced BRT after 6 weeks and suggested that patients should be permitted to drive after this time. However, it is not clear whether their results may be extrapolated to patients driving a car with a coupler because Pierson et al. used a test device with two pedals and permitted braking with the right or the left foot. Additionally, 13 of their patients had undergone simultaneous bilateral TKA.

Given the high incidence of knee osteoarthritis [14] which is intensified by an aging population [15], the increasing numbers of TKA being performed [1-3], and the restrictions of the above mentioned studies [7,10] BRT before and after TKA has become a highly relevant issue. We hypothesized that, on longitudinal comparison, pre-operative (pre-op, 1 day before surgery), post-operative (post-op, 2 weeks post surgery) and follow-up (FU, 8 weeks post surgery) values for BRT would show significant differences among patients with TKA. Specifically, we hypothesized that patients with right-sided TKA show a significant increase in BRT from pre-op to post-op (hypothesis 1) and a significant decrease in BRT from post-op to FU (hypothesis 2). The same was hypothesized for patients undergoing left-sided TKA (hypothesis 3 & 4, respectively). Additionally it was hypothesized that the BRT of patients after TKA is significantly higher than that of healthy controls at each time point (hypothesis 5).

## Methods

### Participants

**Figure 1:**
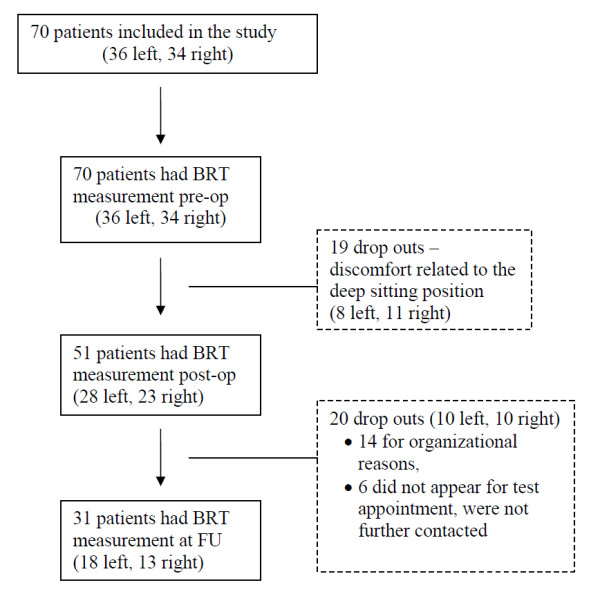
**Flow of patients/drop-outs after inclusion in the study**.

In 70 consecutive patients awaiting TKA of the left or right knee we assessed BRT preoperatively using the custom-made apparatus described below. The study was confined to patients with a driver's license. Exclusion criteria were as follows: a) a metabolic or neurological disease that might affect sensorimotor performance (e.g. diabetes, Parkinson's disease), b) patients reporting pain of musculoskeletal origin other than the knee and c) patients without adequate language skills. Of the 70 Patients recruited from October 2001 to May 2002 19 dropped out prior to the post-op test and further 20 dropped out before the FU test (see Figure 1 for details).

TKA was performed in the left leg in 18 patients and in the right leg in 13. A cemented total knee prosthesis (Kinemax, Stryker, Kalamazoo, Michigan) was implanted via a standard medial parapatellar approach. The patients underwent a standard rehabilitation program after surgery, consisting of continuous passive motion, and active and passive exercise under the guidance of a physiotherapist. Subsequently most of the patients attended an outpatient rehabilitation program. One patient had a wound dehiscence which was treated by skin grafting.

The control group consisted of healthy subjects who also had a driver's license, at least 10 years of driving experience, and no history of knee pathology. Characteristics of the participants are shown in Table 1.

**Table 1:** Participant demographics, descriptive statistics on BRT of TKA patients and proportion of patients above or below the threshold of 700 ms of BRT

		Right-sided TKA	Left-sided TKA	Controls
		(n = 13)	(n = 18)	(n = 31)
**Age (mean (SD)) [y]**		65.9 (12.4)	65.7 (8.9)	52.0 (7.7)
**Female [n]**		9	8	19
**Male [n]**		4	10	12

**BRT pre-op (mean (SD)) [msec]**		664 (64)	632 (46)	
**BRT post-op (mean (SD)) [msec]**		674 (65)	642 (56)	
**BRT FU (mean (SD)) [msec]**		643 (55)	626 (50)	

**BRT pre-op**	**< 700 ms [n]**	9	17	
	**> 700 ms [n]**	4	1	

**BRT post-op**	**< 700 ms [n]**	9	14	
	**> 700 ms [n]**	4	4	

**BRT FU**	**< 700 ms [n]**	11	17	
	**> 700 ms [n]**	2	1	

The changes in the proportions of patients were found to be non-significant (Cochran test).

BRT: Brake Response Time

TKA: Total Knee Arthroplasty

pre-op: preoperative (1 day before surgery)

post-op: postoperative (2 weeks after surgery)

FU: follow-up (8 weeks after surgery)

SD: standard deviation

The tests were conducted according to the Declaration of Helsinki [16] and informed consent was obtained from all subjects prior to participation. The study protocol was approved by the local ethics committee.

### Materials

**Figure 2:**
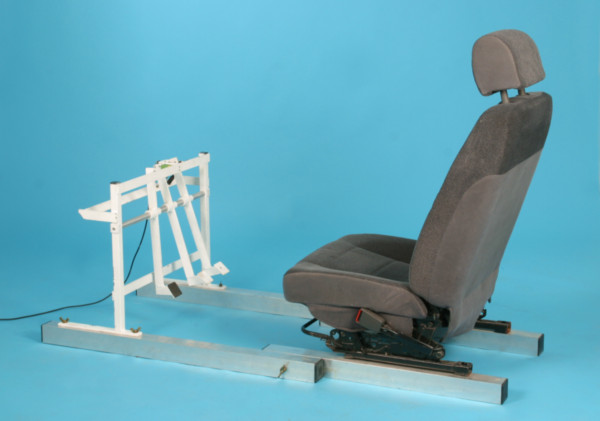
**Custom-made apparatus to measure brake response time**.

**Figure 3:**
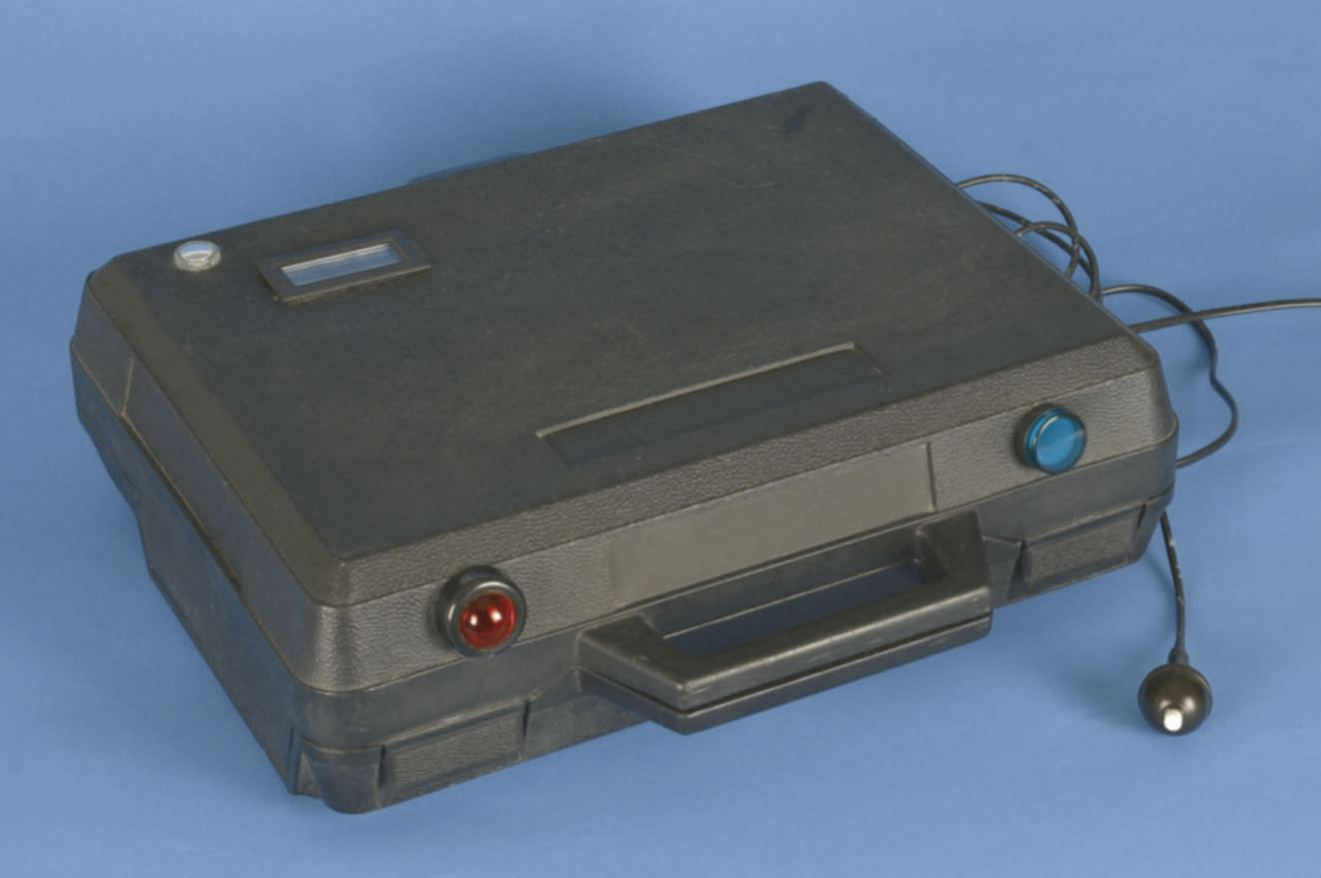
**The external suitcase containing the logic gate electronics, the trigger, the green and red lamp, and the clock**.

Based on apparatuses described and validated in the published literature [6,9] we devised an experimental device to measure BRT (Figure 2 and 3). An adjustable car seat was fixed on a frame with hanging pedals mounted on rubber damped pivots. The inclination of the seat, the head rest and the seat-pedal distance were adjusted according to previous investigations [17] such that they resembled the patient's usual driving position. An external suitcase containing the logic gate electronics, a green and a red lamp were positioned on a table at a fixed distance in front of the frame.

The experimental set-up has been described in a recent study [18].

### Procedure

When the accelerator was completely depressed the green lamp shone, indicating that the patient did not 'drive' in a 'ready-to-brake fashion'. After 5 to 10 seconds the same investigator pushed an external trigger invisible to the patient, which activated the red lamp and the electronic time clock. Subjects were instructed to apply the brake 'as quickly as possible' with the right foot when the signal appeared. The time interval between the appearance of the signal and the subject operating the brake was measured, displayed on the digital clock in milliseconds, and taken as BRT. The brake was always operated with the right leg while the left foot rested on the coupler pedal. After the patient had become familiar with the apparatus (three trial runs) BRT was measured ten times by this procedure. An interval of 5 seconds was permitted between every two measurements. All participants were given the same standardized instructions. In view of the adherence to previous study protocols [6], reproducibility tests were deemed unnecessary.

In the patient groups, the test was conducted pre-op, post-op and at FU. Control subjects were tested only once, by the same procedure described above.

### Data analysis

For each test series the two lowest and two highest trials were discarded. The average of the remaining six values was taken as the individual's BRT and processed using the Statistical Package for Social Sciences (SPSS-Norusis/SPSS Inc., Chicago, IL, USA). BRT data were not normally distributed and therefore regarded as 'ordinal' variables. Median (Md) and interquartile ranges (IQR) were calculated as descriptive statistics. For inferential statistics, the Friedman test was applied to test for longitudinal differences (dependent variables) in BRT. P-values < 0.05 were deemed statistically significant. In case of significant results, Wilcoxon tests were used for post-hoc analyses (pre-postop comparison, postop-FU comparison); p-values < 0.025 were defined as being statistically significant. Cross-sectional testing (independent variables) was performed with Mann-Whitney-U tests at an alpha level of 0.05. In addition, we calculated the proportions of patients below or above a BRT threshold of 700 ms and determined significance in preop-postop-FU changes using the Cochran test. The threshold of 700 ms was chosen because several road authorities recommend absolute maximum BRT values between 700 and 1500 msec; we selected the most conservative value [19-21].

## Results

Of the 70 patients recruited, 19 dropped out prior to the post-op test and further 20 dropped out before the FU test (see Figure 1 for details). The mean age of the remaining 31 patients - 17 women and 14 men - was 65.7 years (SD 10.2 years). The 31 controls consisted of 19 women and 12 men with a mean age of 52 years (SD 7.7)

There were no significant increases between pre-op and post-op BRT values for patients who had undergone left- or right-sided TKA. From post-op to FU, there were significant reductions in BRT for patients after right-sided TKA (668 to 642 ms; p = 0.001) and for those after left-sided TKA (624 to 607 ms; p = 0.003) (Table 2). Only minor changes were seen in the proportions of patients below or above the threshold of 700 ms (Table 1). The longitudinal changes in the proportions of patients above or below the 700-ms threshold were found to be non-significant (Cochran test).

**Table 2:** Longitudinal and cross-sectional comparison of BRT in patients after right- and left-sided TKA (pre-op: preoperative; post-op: postoperative; FU: follow-up)

	pre-op				post-op				FU	Friedman Test
**Left-sided TKA**	613 (82)	←	0.058	→	624 (101)	←	0.003	→	607 (76)	0.004
										
(n = 18)	↑				↑				↑	
	0.097				0.128				0.246	
	↓				↓				↓	
										
**Right-sided TKA**	657 (112)	←	0.463	→	668 (112)	←	0.001	→	642 (97)	< 0.001
										
(n = 13)										

Data in frames: brake response time as median (interquartile range) [msec]

Data outside frames: p - values of longitudinal (α = 0.025) and cross-sectional (α = 0.05) comparisons

(Among patients who accomplished only pre-op and post-op measurements (10 right and 10 left), significant increases in BRT were registered for patients after right-sided TKA (669 to 696 ms; p = 0.022) and those after left-sided TKA (658 to 690 ms; p = 0.028)).

The BRT of controls was 487/116 (Md/IQR) - which was significantly better than the BRT of patients with right- or left-sided TKA at all three time points (p < 0.001 respectively).

## Discussion

Neither right- nor left-sided TKA led to a statistically significant increase in BRT from pre-op to post-op. This was supported by our comparisons with the pre-op BRT value as reference as well by the analysis concerning the absolute threshold of 700 ms. The threshold of 700 ms was chosen because a number of road authorities recommend absolute maximum BRT values between 700 and 1500 msec; we selected the most conservative value [19-21]. Our findings do not support our hypotheses 1 and 3. It appears unnecessary to advise patients who have undergone right- or left-sided TKA to refrain from driving beyond 2 weeks after surgery. The small and non-significant increase in median BRT after surgery (11 msec for right- as well as left-sided TKA) would signify a difference of 0.3 m stopping distance at a speed of 100 km/h (27.78 m/s or 62.14 mph).

Significant decreases in BRT from post-op to FU were ascertained for patients who had undergone right- or left-sided TKA when analyzed using Friedman and Wilcoxon tests. This supports our hypotheses 2 and 4. Interestingly, no such significant decreases were found when the data were analyzed with regard to the BRT threshold of 700 ms but that analysis was regarded only as secondary.

Our recommendation of imposing no driving abstinence beyond 2 weeks after surgery for patients with *left-*sided TKA is similar to that expressed by Spalding et al. [7] and Marques et al. [22]. Spalding et al. suggested that patients with left-sided TKA need no postoperative driving abstinence as long as they are strong enough to press the coupler pedal [7] and Marques et al. recommended driving abstinence for 10 days post-surgery in cases of automatic transmission [22].

However, our recommendation of imposing no driving abstinence after two weeks post surgery for patients with *right-*sided TKA is in contrast to previously expressed conclusions: Spalding et al. recommend 8 weeks [7], Pierson et al. suggest 6 weeks [10] and Marques et al. advise 30 days [23] of driving abstinence after right-sided TKA. The potential reasons for the broad inconsistencies among the different studies deserve further discussion: Pierson et al. examined 13 cases of bilateral, 12 cases of right-sided, and six cases of left-sided TKA, and suggested six weeks of driving abstinence. It should be noted that simultaneous bilateral TKA is not routinely performed at the majority of institutions. Moreover, their test device had only two pedals and braking with the right or the left foot was permitted. Spalding et al. used a more complex test procedure, during which two lines on a screen had to be aligned before braking. As BRT is known to be influenced by the complexity of the task [23], the above mentioned differences between the studies may have been due to differences in the test procedure.

Controls had BRT of 487/116 (Md/IQR) which was significantly better than the BRT of patients with right-sided TKA at all 3 time points (p < 0.001). The same statistical results were obtained on comparison with left-sided TKA. The difference is most likely due to several factors, of which pain and muscle weakness due to reflex inhibition must be considered [24]. These factors prolong the time taken to transfer the foot as well as generate sufficient force on the brake pedal.

Several additional factors such as neurological reaction time and foot transfer time may influence BRT and act as confounders. Monitoring visual acuity, which is important aspect of an individual's reaction to visual stimuli, may have yielded interesting data. The effects of general anesthesia on driving ability have been studied. However, the impairing effect of general anesthesia does not persist for longer than 24 hours. The kinematics of the leg might also be a significant aspect. During acceleration the forefoot is on the throttle and the heel rests on the floor. It is possible to complete the process of braking by flexing the right leg at all major joints and lifting the entire foot from the floor, then adducting the leg towards the brake and again extending the leg. It is also possible to lift just the right *fore*foot by dorsiflexion of the ankle, adduct the leg at the hip using the heel as a pivot, and then activate the brake by plantarflexion of the foot. Scott et al. [17] measured BRT in healthy subjects and registered the fastest BRT in subjects who solved the task by only moving the foot. According to the authors, that kinematic strategy signifies less motor unit recruitment and may hence serve as an advantageous factor. The study of braking kinematics may well be addressed in future studies. The ergonomics of pedals must be considered in the context of foot travel time [17,25]. Pedals may 'hang' from the bulkhead - as in our test apparatus - or 'stand' on the floor, or be a combination of the two types. The published literature reveals no preferences with regard to hanging or standing pedals. Official regulations on this point are not known to exist. Therefore our test device could be regarded as a realistic simulation of the actual situation.

We acknowledge the following limitations of the present study, which may also be viewed as recommendations for future studies: of foremost significance is the high drop-out rate in our population. We investigated whether our drop-outs differed from patients who had undergone 3 measurements and found no significant difference in age, gender, baseline BRT or proportion with left or right TKA. We did not determine the individual components of BRT, such as reaction time and movement time. We further acknowledge that we did not succeed in age-matching of the control group. This was difficult because we tried to include controls free of knee complaints. More frequent post-op tests (such as 1 week, 2 weeks, 4 weeks, 6 weeks, etc.) would have yielded more information about the recovery of BRT. We did not determine the level of pain or the analgesic dose. As pain influences muscle performance - and therefore BRT - via reflex inhibition [24], we recommend the use of visual analogue scales for pain in future studies. Furthermore, it might be of interest to perform BRT subgroup analyses for patients with ongoing postoperative knee pain or swelling. It might be speculated whether fatigue or sustained knee flexion - as during real driving - could have an impact on BRT. Therefore, future studies should include tests after sitting in the seat for a longer period. Last but not least, legal implications were beyond the scope of the investigation and were therefore not addressed.

## Conclusions

This small study of patients undergoing unilateral TKA could not detect a significant increase in BRT two weeks after surgery and therefore could not support imposing driving restrictions beyond this time period. Further research is warranted to identify possible subgroups of patients at risk of being above recommended BRT thresholds at this time.

## List of abbreviations used

BRT: brake response time; Pre-op: preoperative (1 day before surgery); Post-op: postoperative (2 weeks after surgery); FU: follow-up (8 weeks after surgery); IQR: interquartile range; MD: Median; SD: standard deviation; TKA: total knee arthroplasty

## Competing interests

The authors declare that they have no competing interests.

## Authors' contributions

MKe carried out the BRT measurements. CH developed the test apparatus. MCL performed the statistical calculations, drafted the manuscript, and revised the text. MKr developed the design of the study and helped to draft the manuscript. CK and DN contributed to the revision of the manuscript. All authors read and approved the final manuscript.

## Pre-publication history

The pre-publication history for this paper can be accessed here:

http://www.biomedcentral.com/1471-2474/11/267/prepub
